# Drying–Wetting Correlation Analysis of Chloride Transport Behavior and Mechanism in Calcium Sulphoaluminate Cement Concrete

**DOI:** 10.3390/ma17184600

**Published:** 2024-09-19

**Authors:** Lingbo Wang, Hangjie Zhou, Songsong Lian, Xudong Tang

**Affiliations:** 1 College of Civil Engineering and Architecture, Zhejiang University, Hangzhou 310058, China; lbwang@zju.edu.cn (L.W.); 22312066@zju.edu.cn (H.Z.); 2 School of Civil Engineering, NingboTech University, Ningbo 315100, China; lianss@nbt.edu.cn; 3 School of Civil and Transportation Engineering, Ningbo University of Technology, Ningbo 315211, China

**Keywords:** calcium sulfoaluminate cement, chloride erosion, drying–wetting cycles, correlation, microanalysis

## Abstract

In response to rising CO_2_ emissions in the cement industry and the growing demand for durable offshore engineering materials, calcium sulphoaluminate (CSA) cement concrete, known for its lower carbon footprint and enhanced corrosion resistance compared to Ordinary Portland Cement (OPC), is increasingly important. However, the chloride transport behavior of CSA concrete in both laboratory and marine environments remains underexplored and controversial. Accordingly, the chloride ion transport behaviors and mechanisms of CSA concrete in laboratory-accelerated drying-wetting cyclic environments using NaCl solution and seawater, as well as in marine tidal environments, were characterized using the rapid chloride test (RCT), X-ray diffraction (XRD), mercury infiltration porosimetry (MIP), and thermogravimetric analysis (TGA). The results reveal that CSA concrete accumulates more chloride ions in NaCl solution than in seawater, with concentrations 2–3.5 times higher at the same water–cement ratio. Microscopic analysis indicates that calcium and sulfate ions present in seawater facilitate the regeneration of ettringite, thereby increasing the density of the surface pore structure. The hydration and repair mechanisms of CSA concrete under laboratory conditions closely resemble those in marine tidal conditions when exposed to seawater. Additionally, this study found that lower chloride ion concentrations and pH levels inhibit the formation of Friedel’s salt. Therefore, laboratory experiments with seawater can effectively simulate CSA concrete’s chloride transport properties in marine tidal environments, whereas NaCl solution does not accurately reflect actual marine conditions.

## 1. Introduction

The substantial quantity of Portland cement utilized in concrete construction results in significant CO_2_ emissions, accounting for approximately 10% of global anthropogenic greenhouse gases [1]. There is a growing recognition of the need to reduce CO_2_ emissions in the cement industry. Currently, optimization of cement production equipment [2], development of new cementitious materials [3,4,5], and partial replacement of clinker with supplementary cementitious materials (SCM) such as municipal solid waste incineration bottom ash(MSWIBA) [6] represent a few of the main avenues for reducing CO_2_ emissions from cement production. In comparison to Ordinary Portland Cement (OPC), calcium sulfoaluminate (CSA) cement, a cement developed by the China Academy of Building Materials Research (CABMR), exhibits low-carbon and corrosion-resistant properties. It has recently garnered increasing attention from both the academic community and the engineering community [7,8,9]. There are several key components in CSA cement including anhydrous calcium sulfoaluminate (3CaO·3Al_2_O_3_·CaSO_4_), belite (2CaO·SiO_2_), and gypsum (CaSO_4_·2H_2_O) [10], and its main hydration products are ettringite (AFt), aluminum hydroxide (AH_3_), and calcium silicate hydrate (C-S-H) gel. The performance characteristics of CSA cement include fast setting and fast hardening, high early strength, micro-expansion, frost resistance, corrosion resistance, and low alkalinity [11,12]. The aforementioned performance characteristics render CSA cement a versatile material, suitable for a multitude of applications in special environments, such as low temperatures and marine conditions.

In the marine environment, particularly in the tidal zone, where oxygen and moisture are plentiful, concrete is particularly susceptible to the erosive effects of chloride ions [13]. A number of studies have demonstrated that CSA cement exhibits superior corrosion resistance, particularly with regard to sulfate erosion, which makes it highly resistant to seawater erosion [14,15,16]. However, the chloride transport mechanism of CSA cement concrete remains a subject of debate. Zhao et al. [17] demonstrated that CSA cement concrete exhibits superior resistance to chloride erosion after freeze–thaw cycles compared to ordinary silicate cement concrete. Other studies have indicated that the low alkalinity of CSA cement and the low binding capacity of chloride ions are detrimental to the chloride ion erosion resistance of CSA cement. Paul et al. [18] argued that the primary hydration products of CSA cement, calcite, and alumina hydroxide lack the capacity to chemically bind chloride ions. Thomas et al. [19] revealed, through two years of marine exposure experiments, that the total chloride ion concentration within CSA cement concrete was higher than that of OPC concrete, and that Friedel’s salt was not observed in CSA cement concrete. In order to further investigate the mechanism of chloride erosion resistance of CSA cement concrete, a large number of marine exposure experiments are required. However, since experiments in actual marine environments often require years or even decades-long observations, researchers often use artificially simulated environments in order to conduct accelerated experiments in the laboratory, which effectively shortens the research cycle [20].

Laboratory-accelerated experiments hasten the corrosion process and deterioration of concrete by constructing more severe experimental conditions than those typically encountered in the normal service environment of concrete. This approach allows for the accumulation of a large amount of experiment data in a relatively short period of time, which can be used to evaluate the corrosion resistance of materials and select the most appropriate materials for a given application [21]. In the laboratory, a chloride salt attack on concrete is typically simulated using salt solutions, salt spray environmental chambers, and other similar apparatus [22]. Zhang et al. [23] conducted field tests and laboratory simulations to study the time variability and similarity of chloride ion concentration peaks in concrete. The results showed that the chloride ion concentration peak after 120 days of exposure in a simulated environment could effectively reflect the peak concentration observed in the field after 360 days. Laboratory-accelerated experiments must meet several conditions: a high acceleration ratio, corrosion mechanisms consistent with marine exposure experiments, and good controllability and repeatability of experiment results [24]. However, it is difficult and inaccurate to realize the above conditions, and there is still a big difference between the accelerated experiments in the laboratory and the actual marine environment. Therefore, it is necessary to compare the results of accelerated experiments in the laboratory with those of experiments in the marine environment in order to improve the accuracy of accelerated experiments in the laboratory.

In this study, the characterization parameters of chloride ion transport in CSA cement concrete under chloride salt drying–wetting cycles, seawater drying–wetting cycles, and marine exposure environment in the tidal zone were investigated, respectively. The chloride ion transport mechanism was analyzed using microscopic characterization means such as XRD and SEM. The differences in chloride erosion in these three environments were then correlated to compare chloride transport mechanisms under different environmental conditions. The goal is to make laboratory-accelerated drying–wetting cyclic experiments reflect the experimental results in the marine environment so that they have predictive value in practical applications.

## 2. Materials and Methods

### 2.1. Materials

**Figure 1:**
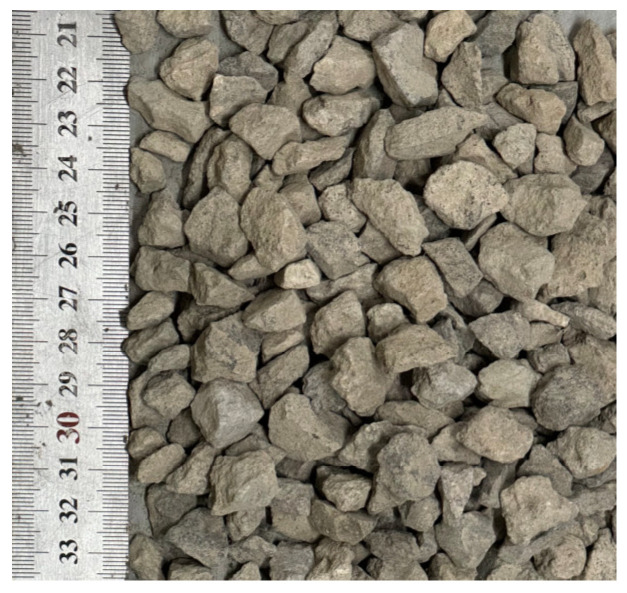
Coarse aggregate used in the experiment. (unit: cm).

In this study, 42.5-grade calcium sulfoaluminate (CSA) cement with a density of 2519 kg/m^3^ and a specific surface area of 455 m^2^/kg was used with the chemical composition shown in Table 1. The coarse aggregate used was limestone with a diameter of 5–25 mm and a density of 2740 kg/m^3^, as illustrated in Figure 1. The fine aggregate was medium river sand with a fineness modulus of 2.4 and a density of 2620 kg/m^3^.

The water-reducing agent used in the experiment was polycarboxylic acid superplasticizer, which was in powder form and had a water reduction rate of 25%. In addition, analytically pure boric acid (AR, ≥99.9%) produced by Shanghai Aladdin Biochemical Technology Co., Ltd. (Shanghai, China) was used as a retarder to improve the workability of concrete.

### 2.2. Mix Design

Three different water–cement ratio conditions corresponding to three groups were established in this study. Boric acid and polycarboxylic acid superplasticizer were both used at a dosage of 0.2% by weight of the cement. The proportion of calcium sulfoaluminate cement concrete under different water–cement ratio conditions is shown in Table 2.

### 2.3. Sample Preparation

Concrete specimens were prepared using a concrete mixer. Raw materials such as sand, stone, cement, and chemical additives were first added to the mixer and stirred for one minute to mix the raw materials well, then mixing water was slowly added while stirring until exhausted. The total mixing time was 3 min. Before the formal test, under the premise of not changing the water–cement ratio, the pre-test was carried out by adjusting the sand rate, water-reducing agent mixing, etc., so that the slump of the fresh concrete reached the interval of 100 ± 10 mm, to ensure that the fresh concrete would have better working performance and molding quality. The slump test was performed on the fresh concrete obtained after mixing, and when the slump was close to the target value, the concrete was placed in a 100 mm × 100 mm × 100 mm mold and fully vibrated. The mold was covered with plastic film and removed after 6 h. The specimens were then demolded and placed in a standard curing room (temperature: 20 ± 2 °C; humidity: 98 ± 2%).

**Figure 2:**
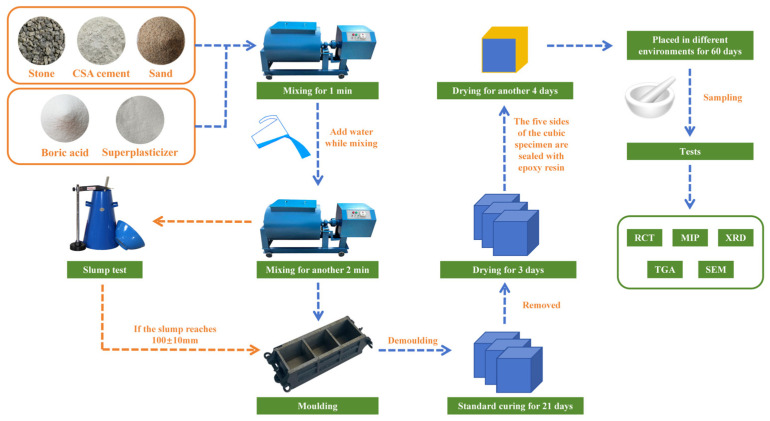
Schematic diagram of the sample preparation process.

To ensure one-dimensional unidirectional transport of chloride ions in the accelerated drying–wetting cyclic tests, concrete specimens were removed and dried at room temperature for 3 days after the standard curing period of 21 days. Subsequently, one side of the specimen was selected as the corrosion surface, and the other five surfaces were sealed with epoxy resin, and the specimen was sealed and allowed to dry until day 28 to ensure that the epoxy resin was fully cured. After treatment, the concrete specimens were placed in different environments for curing and sampling after 60 days. The whole process of sample preparation can be seen in Figure 2.

### 2.4. Curing Condition

#### 2.4.1. Marine Tidal Environment

The actual service environment in this study is the tidal zone of Zhairuoshan Island (N29.95°, E122.09°) in the southern sea area of Zhoushan City, Zhejiang Province. The island has a north-south geomorphology with a length of 2.26 km, a width of 1.01 km, a total land area of about 2.34 km^2^, and a coastline of 7.27 km [25]. The salinity of the seawater is 2.5% with an average water temperature of about 17 °C. The specific composition is given in Table 3 and the main corrosive ions are Cl^−^, SO_4_^2−^, and Mg^2+^. In addition, the tidal type in this area is an irregular semi-diurnal tidal pattern.

**Figure 3:**
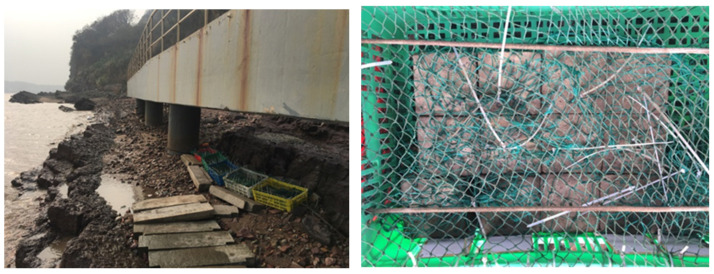
Sample preparation cured in a marine tidal environment.

In this area, the specimens are completely submerged by seawater at high tide and exposed to air at low tide. The specimens are placed in perforated plastic baskets, then enclosed in fishing nets and secured between two piers with thick ropes to ensure the stability and safety of the specimens during the marine exposure test, as seen in Figure 3.

#### 2.4.2. Laboratory-Accelerated Drying–Wetting Cyclic Environment

Based on the data from the tidal zone of Zhairuoshan Island, the laboratory-accelerated drying–wetting cyclic system designed in this study is detailed in Table 4, and the experiment was conducted using CARB-LSB/2 automatic drying–wetting cyclic testing machine.

To simulate the twice-daily tidal cycle in the tidal zone, each drying–wetting cycle was set to include a 6-h wetting phase and a 6-h drying phase. In view of the concentration of chloride ions in seawater and the erosive effect of a single ion, a NaCl solution with a concentration of 2.5% was selected as the corrosive solution, which has a similar chloride ion content to seawater. Anyway, in order to accurately simulate the marine environment, this experiment also used seawater with multiple ion compositions collected from the tidal zone of Zhairuoshan Island as the corrosive solution. In the experiment, both corrosion solutions were replaced every 15 days to reduce the experimental error caused by the variation in ion concentration. 

Ambient temperature as well as drying–wetting cycle conditions have a significant effect on chloride ion transport in concrete in the tidal zone. Since the accelerated drying–wetting cycle in the laboratory of this study was similar to that in the tidal zone, ambient temperature was used as the accelerating factor for the experiment.

It has been shown [26,27] that the main hydration product of CSA cement concrete, ettringite, begins to dehydrate and decompose at 80 °C. Meanwhile, under direct solar radiation, the temperature of the concrete surface can reach 40–60 °C [28]. In order to ensure the acceleration effect without changing the chloride ion transport mechanism, 60 °C was selected as the accelerated drying temperature in this experiment.

To facilitate the analysis of the experimental results, the concrete specimens were numbered, where C represents CSA cement concrete specimens, I and O represent the laboratory environment and the marine environment, respectively, and N and S represent NaCl and seawater, respectively. CIS refers to CSA cement concrete specimens soaked in seawater under laboratory conditions, CIN refers to CSA cement concrete specimens soaked in NaCl solution under laboratory conditions, and COS refers to CSA cement concrete specimens soaked in seawater in a marine environment. The number before the dash represents the water–cement ratio as a percentage, while the number after the dash indicates the number of days of the exposure experiment.

### 2.5. Testing Methods

#### 2.5.1. Rapid Chloride Permeability Test

A China Xinyu HDM-1A concrete grinding machine was used to take samples of the first 30 mm of the concrete specimen in layers: the first 6 mm of the specimen at every 1 mm and the second 24 mm of the specimen at every 2 mm. Then, the powder samples of each layer obtained after grinding were sieved with 0.16 mm square hole sieve mesh, and then the sieved samples were stored in sealed bags for subsequent tests.

The determination of the free chloride ion concentration of concrete was carried out by a Rapid Chloride Concentration Tester (RCT), and the instrument used was a Korean DYS DY2501-B type chloride ion tester. The free chloride ion concentration in concrete was determined by a water-soluble extraction method according to the standard procedure in the instruction manual of the instrument. Two grams of concrete powder samples were dissolved in 20 g of water-soluble extraction solution, shaken vigorously for 10 min, and then measured by a chloride ion tester after standing for 24 h.

#### 2.5.2. Pore Structure Test

Mercury intrusion porosimetry (MIP) was used to measure the microscopic pore structure of concrete before and after corrosion. The instrument utilized was a U.S. Mack AutoPore 9510 fully automated mercury porosimeter, capable of measuring pore sizes ranging from 0.1 to 60,000 psia. The experimental procedure was conducted in accordance with the standards set forth in ISO 15901-1, specifically “Evaluation of pore size distribution and porosity of solid materials by mercury porosimetry and gas adsorption—Part 1: Mercury porosimetry”. In this paper, samples were taken from the surface layer of the concrete specimens at a depth of 0–10 mm, ground into 2–3 mm particles, and then placed in a 60 °C oven to dry for 24 h for the experiments.

#### 2.5.3. X-ray Diffraction (XRD)

X-ray diffraction (XRD) was used to analyze the mineral composition of the concrete before and after corrosion. The cement mortar on the surface layer of the concrete specimens (with the coarse aggregate removed) was collected and placed in an oven at 60 °C for 24 h. The dried samples were ground, and the ground powder was sieved through a 0.16 mm square mesh sieve and stored in a sealed bag. The samples were analyzed using a D8 advance instrument (manufacturer: Bruker, Germany) in Bragg–Brentano geometry with Cu Kα (λ = 1.54 Å) radiation operating at a voltage of 40 kV and a current of 40 mA. The samples were scanned in the 2θ range from 5 to 89° at a scan rate of 0.02°. The experimental procedure followed the guidelines outlined in ASTM C1365-18.

#### 2.5.4. Thermogravimetric Analysis (TGA)

The tests were conducted using thermogravimetric analysis (TGA) for the major mineral content of concrete before and after corrosion, with sample preparation for TG-DTG consistent with XRD. The samples were analyzed using a synchronous thermal analyzer, model TA SDT Q600 from the USA. The test was conducted in accordance with ASTM C1872-18, which outlines the standard test method for the thermal analysis of hydraulic cements by thermogravimetry. The analysis was performed in a nitrogen environment with a heating rate of 10 °C/min and a temperature range of 25 °C to 1000 °C. After the derivation, the DTG curves were obtained, showing a series of peaks corresponding to the decomposition of various hydration phases.

#### 2.5.5. Scanning Electron Microscope (SEM)

Following the crushing of the concrete, surface samples were taken and placed in anhydrous ethanol to halt the process of hydration. They were then placed in an oven at 80 °C to dry to a constant weight. Subsequently, the samples were further crushed to expose fresh sections and fixed with conductive tape with the sections facing upwards. To prevent surface charge accumulation from affecting the imaging quality, the samples were gold sprayed. Finally, the samples were placed into the sample chamber of the SEM for micromorphological observation. The analysis was performed using an HV01–43 instrument (Producer: Sigma, Steinheim, Germany) at a voltage of 5 kV, following the procedure outlined in ASTM C1723-16.

## 3. Results

### 3.1. Correlation of Parameters for Characterizing Chloride Ion Transport

#### 3.1.1. Correlation of Free Chloride Ion Distribution

**Figure 4:**
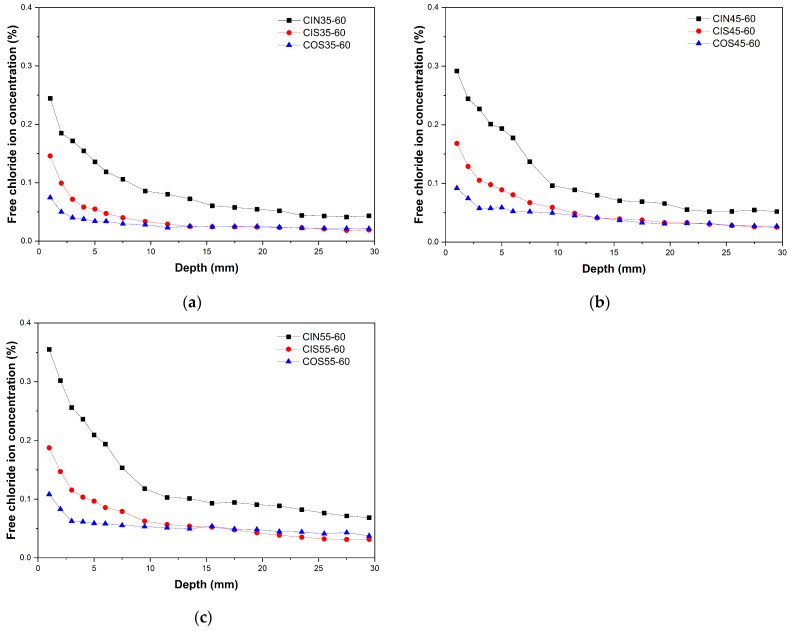
Distribution curves of free chloride ion: (**a**) w/c = 0.35; (**b**) w/c = 0.45; (**c**) w/c = 0.55.

Figure 4 illustrates the free chloride ion distribution curves of CSA cement concrete in three distinct environments: a laboratory chloride salt-accelerated drying–wetting cyclic environment, a laboratory seawater-accelerated drying–wetting cyclic environment, and a marine tidal environment. The curves are presented for three different water–cement ratios.

Figure 4 illustrates the chloride ion distribution characteristics in concretes with different water–cement ratios under various exposure durations in real marine environments. The percentage of chloride presented is based on the weight of the concrete. It can be observed that at each testing age, as the water–cement ratio increases, the concentration of free chloride ions at various depths in the concrete shows a gradual upward trend. This trend is consistent with the patterns observed in samples cured in these three environments. A higher water–cement ratio results in higher porosity in the concrete, increasing the quantity and connectivity of capillary pores, which leads to a reduction in the concrete’s density. During the drying process of wet–dry cycles, this condition accelerates the evaporation of moisture from the concrete surface, making the surface drier and leading to more chloride ion accumulation. Moreover, the increase in the water–cement ratio enhances the chloride ion diffusion coefficient of the concrete, making it easier for chloride ions to diffuse internally, thereby increasing the internal chloride ion concentration. Additionally, with the extension of the exposure time in real marine environments, the chloride ion concentration on the surface of the CSA concrete continues to rise, although the rate of accumulation gradually slows down, while the chloride ion concentration inside the concrete gradually increases due to the diffusion effect.

It can be found that the chloride ion transport rate of CSA cement concrete is increased in the laboratory environment compared to the marine tidal environment. Both environments with different solutions demonstrated a stronger acceleration of chloride ion transport on the surface of the concrete than in the interior. CSA cement generates a large amount of ettringite to fill the pores during the hydration process, which promotes the rapid development of strength [15]. In the context of high-temperature-accelerated drying–wetting cycles, surface layers of ettringite can be carbonized by atmospheric CO_2_ or converted to other phases, such as AFm (Alumina Ferric monosulfate), due to the elevated temperatures involved. The decomposition of hydration products in the surface layer may be the primary factor responsible for the observed increase in chloride ion concentration, which in turn leads to an increase in porosity and the coarsening of the pore structure of the surface concrete. The capillary adsorption effect of the drying–wetting cycle facilitates the transport of moisture-containing chloride ions into the pores on the surface layer of concrete. The diminished impact on chloride transport capacity within the concrete can be attributed to the reduced diffusion of chloride ions within CSA-cemented concrete.

The accelerating effect of seawater solution exposure on chloride ion transport in the surface layer of CSA cement concrete in the laboratory-accelerated drying–wetting cycle was found to be weaker than that of chloride salt solution exposure. Furthermore, the chloride ions present within the concrete specimens subjected to the accelerated drying–wetting cycle in the laboratory exhibited values that were comparable to or even lower than those observed in marine environments within the tidal zone. One potential explanation for this phenomenon is that the presence of calcium ions (Ca^2+^) and sulfate ions (SO_4_^2−^) in seawater facilitates the regeneration of ettringite, which enhances the densification of CSA cement concrete and impedes the transport of chloride ions to a certain extent. Additionally, the increase in drying temperature may promote the hydration of cement particles and the regeneration of ettringite.

The combined effect of convection and diffusion results in a peak of chloride concentration at a specific depth from the surface concrete. This period of rising chloride concentration has been utilized in numerous studies as the convection zone. The extent of the convective zone is of significant importance. If it is relatively small, the influence of the convective zone on the chloride ion transport process can be eliminated by a modified Fick’s law. Nevertheless, no discernible phase of rising chloride ion concentration was observed in this study. This suggests that the convection zone of CSA cement concrete is more restricted than previously thought. This phenomenon can be attributed to the strong self-drying effect of CSA cement concrete [29,30]. During the drying phase of the drying–wetting cycle, the surface concrete water evaporates at a faster rate, resulting in a reduction in relative humidity. Additionally, chloride ions accumulate towards the surface layer of the concrete due to capillary adsorption, with this accumulation becoming more pronounced the closer the surface layer is to the concrete. On the other hand, the diffusion coefficient of chloride ions in CSA cement concrete is relatively low, and the diffusion of chloride ions from the surface layer to the interior is slow, which leads to the accumulation of chloride ions in the surface layer being significantly higher than the diffusion rate. Consequently, the peak of chloride ion concentration is observed in the most superficial layer. In conclusion, the convection zones of CSA cement concrete were all set at 1 mm, and the measured chloride concentration of the first 1 mm was utilized as the surface chloride concentration.

#### 3.1.2. Correlation of Surface Chloride Ion Concentration

For CSA cement concretes with the same material composition but different environmental conditions, the ratio of surface chloride ion concentrations represents the magnification of chloride ion concentrations between the laboratory-accelerated drying–wetting cyclic environment and the marine tidal environment. The ratio can be defined as the similarity ratio n of the surface chloride ion concentrations, as illustrated in Equation (1).

n = C_ci_/C_co_ (1)

In Equation (1), C_ci_ and C_co_ are the surface chloride ion concentrations of the laboratory-accelerated drying–wetting cyclic environment and the marine tidal environment, respectively. The results of the exposure experiments in the 60-day chloride salt drying–wetting cyclic environment, seawater drying–wetting cyclic environment, and marine tidal zone were analyzed to determine the surface chloride ion concentrations of CSA cement concrete at each water–cement ratio. The results are presented in Table 5. As indicated by Equation (1), the similarity rate (n_1_) of surface chloride ion concentration can be calculated as shown in Table 6.

Meanwhile, as can be seen from the tables, the stabilized surface chloride ion concentration significantly increases with the rise in the water–cement ratio, which has been observed in all these three environments with varying degrees, where the increase in the water–cement ratio leads to higher porosity in the CSA concrete, resulting in greater accumulation of chloride ions on the concrete surface.

The similarity rate of surface chloride ion concentration between laboratory-accelerated drying–wetting cycle experiment and marine exposure experiment showed a strong correlation. In all three water–cement ratio conditions, the n_1_ values of the specimens exposed to chloride salts were approximately 3.3, while the n_1_ values of the specimens exposed to seawater were approximately 1.8, which was lower than the former. The results presented in Table 6 indicate a certain multiplicative relationship between the surface chloride ion concentration of the laboratory-accelerated drying–wetting cycle experiments and the marine exposure experiments. This value is more affected by the accelerated regime and less affected by the water–cement ratio. Therefore, the results of the laboratory-accelerated drying–wetting cycle experiments are effective in reflecting the characteristics of the surface chloride ion concentration of the CSA cement concrete under the conditions of marine exposure.

Fick’s second law is frequently utilized to describe the diffusion of chloride ions in concrete, though it relies on a set of idealized assumptions [31]. To more precisely capture the behavior and temporal variations of chloride ion concentration on the surface of CSA cement concrete during accelerated wet–dry cycles, this research adopts an exponential model (Equation (2)) for fitting the surface chloride ion concentration [32].

(2) $$C_{s}=C_{s0}\left(1-e^{-\gamma t}\right)$$

In Equation (2), *C_S_* denotes the surface chloride ion concentration (%); *C*_*S*0_ refers to the stabilized surface chloride ion concentration after a certain period, which is also the maximum surface chloride ion concentration (%); *t* stands for time (years); and *γ* is the fitting coefficient. Table 7 and Table 8 present the fitted values of the maximum surface chloride ion concentration *C*_*S*0_ and the similarity rate n_2_ for CSA cement concrete stabilized by accelerated drying–wetting cycles in the laboratory versus marine exposure, respectively. The surface chloride ion concentration in the concrete increases rapidly at the initial stage but slows down gradually at later stages and eventually stabilizes due to the increase in hydration density and the accumulation of chloride ions on the surface. Therefore, the fitted values calculated using Equation (2) can be used to predict the maximum chloride ion concentration on the surface after environmental erosion stabilization.

#### 3.1.3. Correlation of Apparent Chloride Ion Diffusion Coefficient

Table 9 presents the apparent chloride ion diffusion coefficients D derived from actual measurements taken during the 60-day laboratory-accelerated drying–wetting cycle and marine exposure. The table reveals that in all tests, the apparent chloride ion diffusion coefficients increase as the water–cement ratio increases. This is because a higher water–cement ratio decreases the density of the concrete, facilitating chloride ion diffusion. Table 10 displays the similarity rate n_3_ for the apparent chloride ion diffusion coefficients at 60 days. Similar to the similarity rate of surface chloride ion concentration, n_3_ is the ratio of the apparent chloride ion diffusion coefficient from the laboratory-accelerated drying–wetting cycle experiments to that from the marine exposure experiments. When chloride salt drying–wetting cycles are employed, the n_3_ value is around 0.6, and when seawater drying–wetting cycles are used, the n_3_ value is approximately 0.5. This demonstrates that the results from both accelerated methods are strongly correlated with the actual apparent chloride ion diffusion coefficients and are less influenced by the water–cement ratio. In practical engineering projects, the results from laboratory-accelerated drying–wetting cycle experiments can be utilized to predict the apparent chloride ion diffusion coefficients of CSA cement concrete under marine exposure.

Under the same exposure duration, the apparent chloride ion diffusion coefficient of CSA concrete continuously increases with the rise in the water–cement ratio. Moreover, regardless of the environment, the higher the water–cement ratio, the faster the rate of decrease in the apparent chloride ion diffusion coefficient of the CSA concrete.

### 3.2. Correlation of Microstructure

#### 3.2.1. Pore Structure

**Figure 5:**
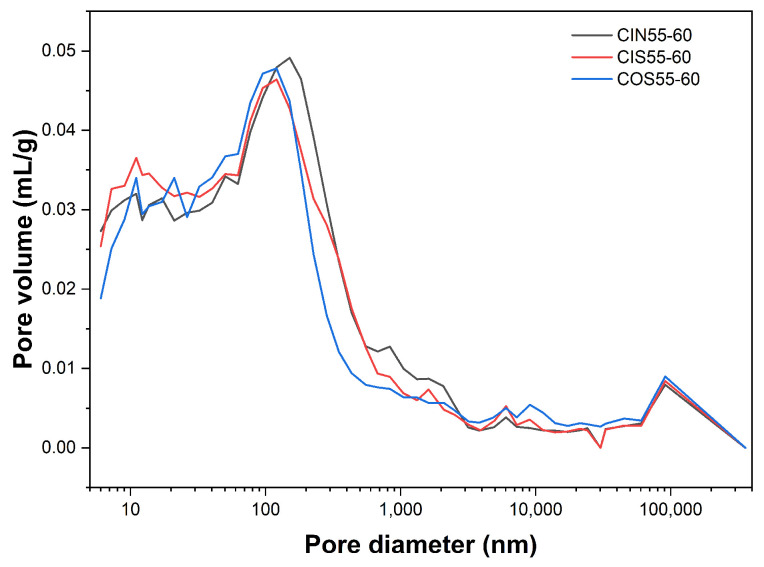
Pore size distribution of CSA cement concrete after 60 days of laboratory-accelerated drying–wetting cycles and marine exposure.

Chloride penetration into the concrete surface primarily occurs through capillary absorption alongside moisture. Consequently, regarding the durability and mechanical properties of concrete, capillary pores larger than 100 nm are considered harmful, while those smaller are categorized as minimally harmful or harmless [33]. Figure 5 depicts the pore size distribution curves of CSA cement concrete subjected to 60 days of laboratory-accelerated drying–wetting cyclic environment and marine exposure environment. The transport of chloride ions at the concrete surface is primarily facilitated by capillary adsorption in the presence of moisture. For the purposes of maintaining the durability and mechanical properties of concrete, capillary pores with diameters exceeding 0.1 μm are generally regarded as detrimental, whereas those with diameters below 0.1 μm are classified as less harmful or harmless. The primary region for capillary adsorption is within the pore size range of 0.1 μm to 100 μm [33,34].

The figure illustrates that in comparison to the laboratory-accelerated drying–wetting cyclic environment, concrete exposed to the marine environment exhibits an increased pore volume around the 10 μm pore size, while the pore volume in the 0.1 μm to 1 μm range significantly decreases. This results in a trend of decreasing average pore size. However, the large pores around 10 μm have a substantial impact on ion penetration, making the chloride ion diffusion coefficient of CSA cement concrete in the marine environment higher than that in the laboratory setting. The CIN55-60 group, in particular, exhibits the most notable increase in pores within the 0.1 μm to 1 μm range, which enhances the capillary adsorption effect of the surface concrete and significantly boosts the chloride ion accumulation rate in this region.

In Figure 5, the area beneath the curve represents porosity, with the peak indicating the most probable pore diameter [35]. Table 11 shows the pore parameters of CSA cement concrete subjected to chloride salt drying–wetting cycles, seawater drying–wetting cycles, and marine exposure.

The average pore size and the most probable pore diameter in the CIN group are significantly larger than those in the CIS and COS groups, while the pore parameters of Group CIS and Group COS are quite similar. This discrepancy can be attributed to the differing exposure solutions. During chloride salt drying–wetting cycles, the presence of sodium and chloride ions increases the solubility of ettringite in CSA cement concrete, leading to its decomposition [36]. This process enlarges the pores [37], resulting in an increase in both the average pore size and the most probable pore diameter. However, in seawater drying–wetting cycles and marine exposure, the calcium and sulfate ions in the seawater react with AFm and aluminum hydroxide (AH_3_) to regenerate ettringite, which fills some of the pores formed by the initial ettringite decomposition.

#### 3.2.2. XRD Analysis

In contrast, higher w/c ratios provide an excess of water, facilitating more complete hydration of the cementitious materials. This results in a higher volume of hydration products, but the increased porosity and larger capillary pores associated with high w/c ratios can negatively impact the mechanical properties and durability of the cement. Excess water can also lead to the formation of more ettringite and monosulfate phases, which may affect the dimensional stability of the material.

**Figure 6:**
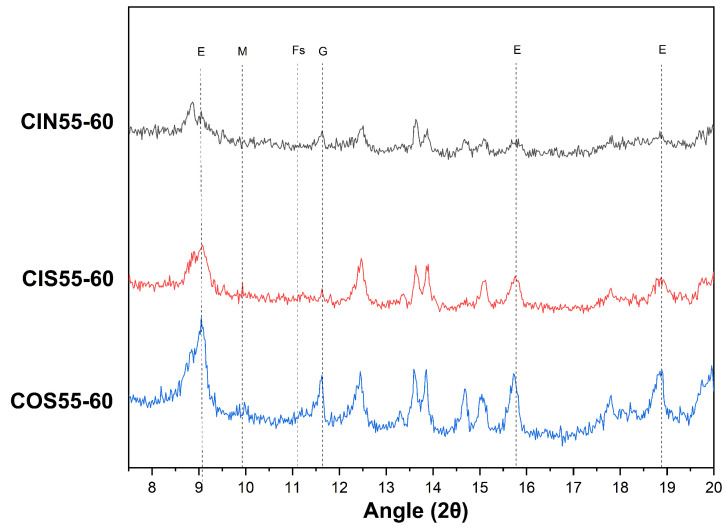
XRD patterns of CSA cement concrete after 60 days of laboratory-accelerated drying–wetting cycles and marine exposure: (E) Ettringite; (M) AFm; (Fs) Friedel’s salt; (G) Gypsum.

Figure 6 compares the XRD patterns of CSA cement concrete subjected to chloride salt drying–wetting cycles, seawater drying–wetting cycles, and marine exposure. Friedel’s salt phase was not observed in the concrete, indicating that the chemical bonding between the hydration products of CSA cement and chloride ions is insignificant. Additionally, the AFm phase was not detected in the spectrum, possibly due to the relatively unstable crystal structure of AFm in cement. Major hydration products of CSA cement, such as ettringite and gypsum, were detected.

The w/c ratio influences the availability of water, which is necessary for the hydration reactions in CSA cement. At lower w/c ratios, the limited water content may restrict the complete hydration of CSA cement particles, leading to a denser microstructure with fewer large pores. This condition favors the formation of ettringite, which contributes to early strength development and durability. However, if the w/c ratio is too low, incomplete hydration may result in unreacted cement particles, yet seawater introduces additional ions that can affect the hydration process and the long-term stability of the CSA cement.

Further examination of Figure 6 reveals that the ettringite peak positions in the CIN55-60 and CIS55-60 samples shift leftward compared to the CIB55-60 sample, with the shift being more pronounced in the CIN55-60 sample. The leftward shift of the peak indicates alterations in the crystal structure of ettringite, including an increase in the lattice constant and distance between atoms within the crystal. These alterations may be attributed to a number of factors, including thermal expansion of the crystal, chemical reactions, the presence of internal defects, or stress [38,39]. It can be inferred that drying–wetting cycles and increased temperatures affect the crystal structure of ettringite, promoting its transformation into AFm and gypsum. Research indicates that the dehydration process of ettringite to AFm is greatly influenced by environmental conditions such as temperature, with high temperatures around 100 °C potentially causing irreversible decomposition of ettringite [40]. Comparison of the ettringite peak shifts in the CIN55-60 group with the other two groups suggests that ettringite is more stable in seawater than in NaCl solution. This is evidenced by the less significant peak shift in seawater, likely due to the Ca^2+^ and SO_4_^2−^ ions in seawater promoting the regeneration of ettringite. Consequently, although seawater drying–wetting cycles also involve high-temperature stages, the ettringite content remains much higher than under chloride salt drying–wetting conditions, resulting in stronger peak intensities.

Table 12 lists the positions, peak intensities, and full widths at half maximum (FWHM) of the main characteristic peaks of ettringite and gypsum. For ettringite, the peak intensity is highest in COS55-60 and lowest in CIN55-60. This observation aligns with the previously discussed mechanism, suggesting that ettringite decomposes during chloride salt drying–wetting cycles, resulting in the destruction of its crystal structure and subsequent carbonation under these conditions. Thus, the peak intensity of ettringite in CIN55-60 is the lowest. 

Moreover, the table reveals that full widths at half maximum (FWHM) of ettringite and gypsum in CIS55-60 are similar to those in COS55-60, suggesting that the crystal sizes of ettringite and gypsum in CSA cement concrete under seawater drying–wetting cycles are similar to those under marine exposure. This is because both drying–wetting cycles take place in seawater, resulting in consistent decomposition and regeneration mechanisms for ettringite.

#### 3.2.3. Thermogravimetric Analysis

**Figure 7:**
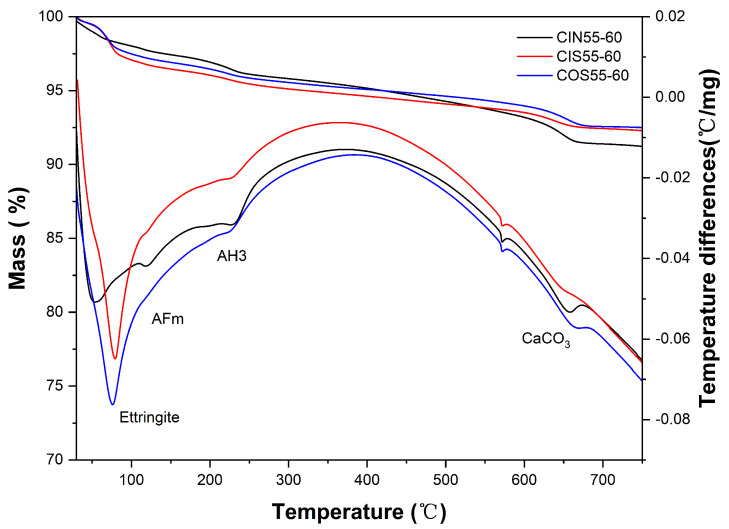
TG-DTG curves of CSA cement concrete after 60 d of laboratory-accelerated drying–wetting cycles and marine exposure.

Figure 7 illustrates the TG-DTG curves of CSA cement concrete under chloride salt drying–wetting cycles, seawater drying–wetting cycles, and marine exposure. The mass loss noted between 80 °C and 110 °C is primarily due to the decomposition of ettringite [41,42]. The mass loss between 130 °C and 140 °C relates to the dehydration of AFm [43,44]. The mass loss from 220 °C to 270 °C is attributed to the decomposition of AH_3_ [45,46], while the mass loss between 600 °C and 700 °C corresponds to the decomposition of calcium carbonate (CaCO_3_) [46,47]. After 60 days of drying–wetting cycles, no endothermic peak for Friedel’s salt was detected, although Friedel’s salt typically shows mass loss peaks around 120 °C and between 180 °C and 450 °C [48,49]. Since the observed peaks are attributed to the dehydration of AFm and the decomposition of AH_3_, we conclude its absence in the tested samples, as confirmed by the XRD results.

After 60 days of drying–wetting cycles, it can be observed that the TG-DTG curves for marine exposure and seawater drying–wetting cycles are more similar in trends. The ettringite endothermic peaks for CIS55-60 and COS55-60 are close, both around 90 °C. Meanwhile, the ettringite endothermic peak for CIN55-60 is significantly shifted to the left and its peak value is reduced, indicating that its crystal structure has changed more than that of CIS55-60 and COS55-60. This is because in the NaCl corrosion solution, the decomposition rate of ettringite is significantly faster than in the seawater corrosion solution, and the decomposed ettringite cannot regenerate. Consequently, the endothermic peak of aluminum hydroxide (AH_3_) shifts to the right, which is caused by the decomposed ettringite.

The mass loss of hydration products under chloride salt drying–wetting cycles, seawater drying–wetting cycles, and marine exposure is shown in Table 13, consistent with the patterns observed in the TG-DTG curves. The ettringite content in the CIN55-60 group is lower than in the other two groups, while the content of aluminum hydroxide and AFm is higher. This indicates that under chloride salt drying–wetting cycles, the decomposition of ettringite is more significant, with aluminum gel and AFm as the main decomposition products. Notably, compared to CIN55-60, the CIS55-60 group has significantly higher ettringite content after the same duration of drying–wetting cycles. This difference may be related to the presence of Ca^2+^ and SO_4_^2−^ ions in seawater, which enhance the stability of ettringite and promote its regeneration. This finding is consistent with the conclusions drawn from previous studies.

Additionally, CaCO_3_ content differs among the three groups. The CIN55-60 group has a higher CaCO_3_ content, which can be attributed to the carbonation and decomposition of ettringite. Ettringite carbonation is a repetitive process highly dependent on the surrounding environment and structural properties [40]. In conditions with insufficient moisture, ettringite carbonation leads to the formation of more hemihydrate gypsum (CaSO_4_·1/2H_2_O) than gypsum (CaSO_4_·2H_2_O). However, in this study, the 60-day drying–wetting cycle provided ample moisture, resulting in gypsum as the main product. Meanwhile, the lower CaCO_3_ content in the CIS55-60 group is attributed to seawater exposure. The continuous supply of SO_4_^2−^ ions enhances the stability of ettringite, inhibiting its reaction with CO_3_^2−^. Given the low permeability of CSA cement concrete [50] and the relatively low atmospheric CO_2_ concentration, carbonation occurs only on the surface of CSA cement concrete.

#### 3.2.4. SEM Analysis

Previous studies have analyzed the microscopic morphology of calcium sulphoaluminate cement concrete under the two different solutions in the laboratory. The results demonstrated that a substantial quantity of ettringite was generated on the surface of the specimens when subjected to both seawater and chloride salt drying–wetting cycles. However, the ettringite formed as a result of the seawater drying–wetting cycles was long and thin, thereby contributing to pore filling in the samples. In contrast, the ettringite under chloride salt drying–wetting cycles was relatively short and thick, and it was also attached with some substances, which were likely related to the decomposed products of ettringite [51]. 

**Figure 8:**
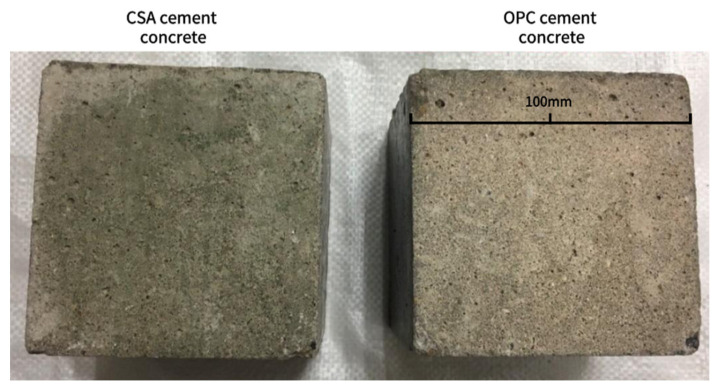
Surface morphology of CSA cement concrete and OPC cement concrete after 60 days of exposure to a marine tidal environment.

**Figure 9:**
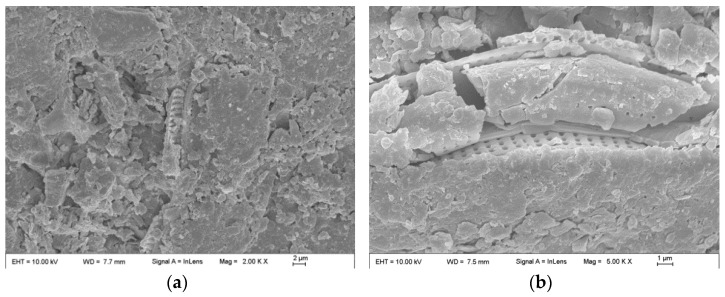

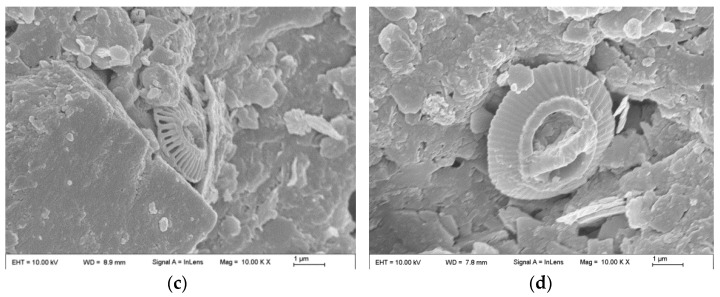
Scanning electron micrographs of CSA cement concrete surfaces in a marine tidal environment: (**a**,**b**) Nitzschia; (**c**,**d**) Cyclotella.

Figure 8 illustrates the macroscopic morphology of CSA cement concrete and OPC cement concrete with an identical mix ratio after 60 days of exposure to a marine tidal environment. It can be observed that the surface layer of CSA cement concrete exhibits a reduced number of macropores and a smoother surface, while the surface layer of OPC cement concrete displays evidence of abrasion, including slight sanding and spalling. This indicates that the pore structure of CSA cement concrete is more dense. Further observation reveals the presence of marine organisms attached to the surface layer of CSA cement concrete. To study this phenomenon in detail, SEM analyses were carried out, as detailed in Figure 9.

The SEM results clearly show that the organisms attached to the surface layer of CSA cement concrete are diatom plankton, such as Nitzschia (double eyebrow diatom) and Cyclotella (small ring diatom), as illustrated in Figure 9. These organisms become tightly embedded in or attached to the pores of the CSA cement concrete. The attachment of marine organisms and their secretions contribute to filling the voids and cracks in the concrete surface, thereby reducing the permeability of the surface layer. This, in turn, helps to inhibit chloride salt erosion and carbonation of the concrete [52,53]. The lower alkalinity of the pore fluid in CSA cement concrete, along with its higher sulfur content [51], makes it more conducive to the adsorption of marine organisms. This further suggests that CSA cement concrete possesses unique advantages in marine environments.

## 4. Discussion

The transport mechanism of chloride ions in concrete primarily involves convection in the surface layer and diffusion in the interior. During the wetting phase, the concrete absorbs the corrosive solution through capillary adsorption until saturation is reached. During the drying phase, the pore water moves to the surface and evaporates through the capillary pore ends exposed to the atmosphere. This evaporation causes the pore water inside to move continuously to the surface under the influence of the moisture gradient, increasing the chloride ion concentration at the surface, which becomes much higher than that in the concrete. This concentration gradient further promotes the diffusion of chloride ions from the surface to the interior. An increase in the water–cement ratio and ambient temperature both enhance the transport of chloride ions. The former increases the porosity of concrete, degrades the pore structure of CSA cement concrete, and accelerates the convection and diffusion of chloride ions. The latter promotes the dehydration and decomposition of ettringite, leading to an increase in the number of harmful pores, thus facilitating the ingress of chloride ions.

In accelerated drying–wetting cycle experiments, carbonation, and higher drying temperatures are considered the primary causes of ettringite decomposition on the concrete surface, forming products such as AH_3_, calcite, gypsum, and AFm. Specifically, ettringite decomposition occurs in two ways: desulfation of ettringite into AFm and gypsum, and carbonation of ettringite into AH_3_, gypsum, and calcite. The above chemical reactions are shown in Equations (3) and (4). Under seawater exposure conditions, the continuous supply of SO_4_^2−^ enhances the stability of ettringite, inhibiting its desulfation and carbonation. Additionally, Ca^2+^ and SO_4_^2−^ in seawater can react with AFm to regenerate ettringite and the corresponding chemical reaction is depicted in Equation (5). The regenerated ettringite can re-fill the concrete’s pores during drying–wetting cycles, refining the pore structure of the concrete surface and hindering chloride ion penetration into CSA samples. This process is similar to the self-healing effect of concrete.

(3) $$C_{3}A\cdot 3CaSO_{4}\cdot 32H_{2}O\to C_{3}A\cdot CaSO_{4}\cdot 12H_{2}O+2\left(CaSO_{4}\cdot 2H_{2}O\right)+16H_{2}O$$

(4) $$C_{3}A\cdot 3CaSO_{4}\cdot 32H_{2}O+3CO_{2}\to 3\left(CaSO_{4}\cdot 2H_{2}O\right)+2Al{\left(OH\right)}_{3}+3CaCO_{3}+23H_{2}O$$

(5) $$C_{3}A\cdot CaSO_{4}\cdot 12H_{2}O+2Ca^{2+}+2SO_{4}^{2-}+20H_{2}O\to C_{3}A\cdot 3CaSO_{4}\cdot 32H_{2}O\;$$

The dehydration process of ettringite into AFm is greatly influenced by environmental conditions such as temperature. In this study, the decomposition temperature of ettringite is significantly reduced, likely due to the immersion of the specimens in corrosive solutions (seawater and NaCl solution). As Zhou et al. [54] pointed out, heating ettringite in water initially causes its dissolution, followed by the formation of more stable AFm at elevated temperatures. It is speculated that the low SO_4_^2−^ concentration in the external solution promotes the dissolution of ettringite, rendering it unstable and thus lowering its decomposition temperature. This aspect requires further investigation. XRD and thermogravimetric analysis revealed that Friedel’s salt was not present in any of the samples. At high chloride ion concentrations, Friedel’s salt can partially replace ettringite, and it decomposes at lower pH values [55]. Therefore, in this study, the lower chloride ion concentration and lower pH [56] prevented the effective chemical bonding between ettringite and chloride ions to form Friedel’s salt. Additionally, the presence of various ions in the seawater also hindered this process.

Building upon the above analysis, the continuous supply of Ca^2+^ and SO_4_^2−^ from seawater promotes the regeneration of ettringite, inhibiting the transport of chloride ions into the concrete. Therefore, the free chloride ion distribution curve for this group is lower compared to the NaCl solution exposure conditions. Additionally, the similarity rates of surface chloride ion concentration and apparent chloride ion diffusion coefficient between laboratory-accelerated drying–wetting cycle tests and marine exposure tests show a strong correlation. Under water–cement ratios of 0.35, 0.45, and 0.55, the similarity rates of surface chloride ion concentration in chloride salt drying–wetting cycles are around 3.3, and the similarity rates of apparent chloride ion diffusion coefficient are around 0.6. In seawater drying–wetting cycles, the similarity rates of surface chloride ion concentration are around 1.8, and the similarity rates of apparent chloride ion diffusion coefficient are around 0.5. Furthermore, the similarity rates are significantly affected by the accelerated drying–wetting cycle regime but are less influenced by the water–cement ratio. Therefore, the results of laboratory-accelerated drying–wetting cycle tests can effectively reflect the characteristics of chloride ion transport parameters in CSA cement concrete under marine exposure conditions, demonstrating their validity and feasibility. However, in marine environments, factors such as biological fouling and mechanical damage are present, which this experiment cannot fully replicate. Additionally, the fitting model is based on a series of assumptions. Therefore, to further validate the effectiveness of laboratory-accelerated tests, longer-term exposure experiments under various environmental conditions are needed, along with the use of different types of corrosive media. A comprehensive evaluation of the durability of CSA concrete is required, and the model needs further optimization to improve its predictive accuracy.

## 5. Conclusions

This study investigates the transport mechanisms of chloride ions in CSA cement concrete under various environmental conditions. By analyzing the similarity rates for the CIN and CIS groups, the results indicate that using seawater in laboratory-accelerated drying–wetting cycle tests provides a more effective simulation of the chloride ion transport characteristics observed in real marine environments compared to the commonly used NaCl solution. The simulation accuracy for surface chloride ion concentration can be as high as 1.89 times greater with seawater, with improved accuracy as the water-to-cement ratio decreases.

Microscopic analysis reveals that under chloride salt drying–wetting cycles, ettringite on the surface of CSA cement is more prone to decomposition, leading to the formation of larger pores and an increased chloride ion transport rate. In contrast, under seawater drying–wetting cycles, the Ca^2+^ and SO_4_^2−^ ions in seawater facilitate the regeneration of ettringite, enhancing the concrete’s density and impeding chloride ion transport, similar to the processes in marine environments. This enhances the simulation’s effectiveness.

Furthermore, the decomposition temperature of ettringite decreases in both environments, likely due to the lower concentration of SO_4_^2−^ in the external solution promoting ettringite dissolution. This conclusion requires further investigation. This study did not observe Friedel’s salt, suggesting that ettringite did not effectively bond with chloride ions, likely due to the lower chloride ion concentration and pH.

This study has certain limitations. The 60-day marine exposure period does not fully capture the deterioration parameters of concrete structures, indicating the need for extended exposure studies (e.g., 2, 5, or 10 years). Additionally, the chloride ion transport model used, based on Fick’s second law, is a basic theory with lower applicability to sulfate-aluminate cement concrete compared to Portland cement systems. Future research should develop a modified theoretical framework for sulfate-aluminate cement concrete based on Fick’s second law.

## Figures and Tables

**Table 1:** Chemical composition of calcium sulfoaluminate cement.

CaO	Al_2_O_3_	Fe_2_O_3_	SiO_2_	MgO	SO_3_	Na_2_O	K_2_O	Loss
44.87%	25.02%	3.09%	9.44%	3.03%	12.07%	0.10%	0.28%	1.46%

**Table 2:** Mix formulation for the preparation of CSA concrete (unit: kg·m^−3^).

Group	w/c	Water	Cement	Sand	Stone	Superplasticizer	Boric Acid
CSA35	0.35	169.5	484.0	611.0	1135.5	0.968	0.968
CSA45	0.45	169.5	376.5	704.5	1149.5	0.753	0.753
CSA55	0.55	169.5	308.0	807.5	1115.0	0.616	0.616

**Table 3:** Composition of seawater in the tidal zone of Zhairuoshan Island (unit: g/L).

K^+^	Ca^2+^	Na^+^	Mg^2+^	Cl^−^	SO^4−^	Br^−^	F^−^
0.519	0.073	7.957	0.856	13.600	1.980	0.011	0.007

**Table 4:** Experimental regime of laboratory-accelerated drying–wetting cycles.

Group	CIN	CIS
Solution	NaCl	Seawater
Cycle duration	12 h	12 h
Wetting temperature	20 °C	20 °C
Drying temperature	60 °C	60 °C

**Table 5:** Measured surface chloride ion concentration of CSA cement concrete at various water–cement ratios.

	w/c = 0.35	w/c = 0.45	w/c = 0.55
CIN	0.2444%	0.2916%	0.3550%
CIS	0.1459%	0.1682%	0.1875%
COS	0.0746%	0.0919%	0.1038%

**Table 6:** Similarity rate of surface chloride ion concentration (n_1_).

	w/c = 0.35	w/c = 0.45	w/c = 0.55
CIN	3.28	3.17	3.42
CIS	1.96	1.83	1.81

**Table 7:** Fitted values of maximum surface chloride ion concentration (*C*_*S*0_).

	w/c = 0.35	w/c = 0.45	w/c = 0.55
CIN	0.2657	0.3997	0.8873
CIS	0.1498	0.1666	0.1827
COS	0.1661	0.2657	0.3058

**Table 8:** Similarity rate of fitted values for maximum surface chloride ion concentration (n_2_).

	w/c = 0.35	w/c = 0.45	w/c = 0.55
CIN	1.60	1.50	2.9
CIS	0.90	0.63	0.60

**Table 9:** Apparent chloride diffusion coefficient D (unit: m^2^/s).

	w/c = 0.35 (10^−11^)	w/c = 0.45 (10^−11^)	w/c = 0.55 (10^−11^)
COS	2.72	3.26	3.92
CIN	1.80	1.95	2.15
CIS	1.46	1.70	1.79

**Table 10:** Similarity rate of apparent chloride ion diffusion coefficients (n_3_).

	w/c = 0.35	w/c = 0.45	w/c = 0.55
CIN	0.66	0.60	0.55
CIS	0.54	0.52	0.46

**Table 11:** Pore parameters of CSA cement concrete after 60 days of laboratory-accelerated drying–wetting cycles and marine exposure (unit: nm).

	Average Pore Size	Most Probable Pore Size
CIN55-60	113.2	151.1
CIS55-60	97.1	120.1
COS55-60	94.4	120.7

**Table 12:** Peak intensities and FWHM of hydration products of CSA cement concrete after 60 days of laboratory-accelerated drying–wetting cycles and marine exposure.

	2θ = 9.1° (Ettringite)		2θ = 11.7° (Gypsum)	
	Peak Intensity	FWHM	Peak Intensity	FWHM
CIN55-60	117	0.294	67	0.092
CIS55-60	211	0.388	36	0.323
COS55-60	415	0.354	209	0.345

**Table 13:** Mass loss of hydration products of CSA cement concrete after 60 days of laboratory-accelerated drying–wetting cycles and marine exposure.

	Mass Loss (wt%)			
	Ettringite (80 °C–110 °C)	AFm (130 °C–140 °C)	AH_3_ (220 °C–270 °C)	CaCO_3_ (600 °C–700 °C)
CIN55-60	0.39	0.26	0.63	1.81
CIS55-60	0.59	0.22	0.49	1.01
COS55-60	0.62	0.22	0.48	1.40

## Data Availability

Data are contained within the article.
